# Effects of Surface Nanotopography and Calcium Chemistry of Titanium Bone Implants on Early Blood Platelet and Macrophage Cell Function

**DOI:** 10.1155/2018/1362958

**Published:** 2018-07-04

**Authors:** Jin-Woo Park, Sang-Hyeob Han, Takao Hanawa

**Affiliations:** ^1^Department of Periodontology, School of Dentistry, Kyungpook National University, Daegu 41940, Republic of Korea; ^2^Institute of Biomaterials and Bioengineering, Tokyo Medical and Dental University, Tokyo 101-0062, Japan

## Abstract

Early responses of blood platelets and immunoinflammatory cells (macrophages) to titanium (Ti) bone implants affect the subsequent biological healing of implants by modulating early tissue healing-microenvironments via the formation of temporary fibrin matrix scaffolds for stem cell migration and production of growth factors and cytokines. This study investigated the effects of nanoscale surface topography and calcium ion (Ca^2+^) modification of Ti surfaces on biocompatibility regulated by blood platelets and macrophages, for the future surface design of Ti bone implants with enhanced early osteogenic capacity. A nanostructured Ti surface with or without Ca^2+^ enrichment was prepared using the hydrothermal treatment. Immediate and early functions of platelets and macrophages modulated by modified Ti surfaces were investigated by morphological observation of platelet spreading and fibrin matrix formation, platelet growth factor release, immunostaining of macrophage phenotypes, and macrophage inflammatory cytokine production. The results showed that surface nanoscale topographical modification of Ti promotes blood platelet activation and suppresses the inflammatory response of macrophages. In addition, surface chemistry modifications with Ca^2+^ enhanced the platelet response-modulating function of the nanostructured Ti surface, which accelerated immediate fibrin matrix formation and platelet-derived growth factor-AB release. Thus, nanotopographical and Ca^2+^ modifications of implant surfaces are expected to be effective approaches that favor the initial phase of wound healing around the Ti bone implants through positive modulation of immediate blood platelet function and early macrophage immunoinflammatory response.

## 1. Introduction

Blood-implant contact is the first biological events that occurs in the wound healing process after the implantation of biomaterials. The immediate early response of platelets to implanted materials affects the biological healing of titanium (Ti) bone implants by causing the release of bioactive molecules and fibrin matrix formation [1–4]. Growth factors and cytokines released by activated platelets recruit macrophages and mesenchymal stem cells (MSCs) onto the implant surface [3, 4]. Studies have demonstrated that positive modulation of the thrombogenic potential of platelets via modification of surface properties of Ti implants induces favorable osseointegration outcomes by the enhancing homing of bone-forming MSCs to implant surface [2, 4, 5]. After implantation, macrophage cells play central role during early wound healing process of implants. Macrophage phenotype development in implanted biomaterials affects wound healing outcome [6–8]. The more dominant expression of the proregenerative (M2) macrophage phenotype compared to the proinflammatory (M1) phenotype favors tissue regeneration, including implant bone growth around the implant, by stimulating the recruitment and osteogenic differentiation of osteoprogenitor cells and suppressing the inflammatory response [6–8].

Recently, nanotopographical and bioactive chemistry have garnered much attention for the surface modification of Ti bone implants [9–11]. Studies have demonstrated that surface chemistry modification using bioactive ions further increases osteogenic capacity of nanostructured Ti implants [9–11]. Studies have also reported that surface chemistry and topographic modification of implants influence the early platelet response, which may subsequently affect macrophage responses and osteogenic cell functions [2, 12–15]. Calcium ions (Ca^2+^) are primarily used for the surface modification of Ti implants to enhance early bone regeneration. Surface Ca^2+^ incorporation promotes early bone formation around Ti implants by promoting the early adhesion and osteogenic differentiation of MSCs [11, 16]. In addition, Ca^2+^ mediates platelet activation [17, 18]. However, it is still unclear whether bioactive ion modification positively modulates immediate blood platelet function at nanorough Ti implant surfaces. We expect that favorable modulations of the immediate and early responses of platelets and immunoinflammatory cells (macrophages) are achieved with modified Ti surface, which consequently promote the replication and osteogenic differentiation of forthcoming MSCs on the implant surface and ultimate implant bone healing.

In this study, the effects of nanostructured and bioactive surface modifications (in this study, Ca^2+^) on the immediate and early responses of platelets and macrophages were investigated, and it was determined if Ca^2+^ modifications of implant surfaces clearly enhance these early biological events at the nanostructured Ti implant surface. To this end, Ti samples with surface nanotopography and Ca^2+^ chemistry were prepared by wet chemical treatment. Then the* in vitro* early healing response was determined by evaluating platelet activation and macrophage phenotype polarization at immediate (within 1 h for platelets) and early (at 24 h for macrophages) time points.

## 2. Materials and Methods

### 2.1. Sample Preparation

Disk-shaped Ti samples 15 mm in diameter and 2 mm thick were made from commercially pure Ti rods (ASTM grade 4). Ti samples were wet-abraded to #1200 grit silicon carbide paper and ultrasonically cleaned in acetone, alcohol, and double-distilled water (control Ti surface). To prepare nanostructured Ti samples with surface Ca^2+^ modification (Nano/Ca surface) and without Ca^2+^ modification (Nanosurface), wet-abraded Ti disks were hydrothermally treated according to a previously described method [11]. Briefly, a pure Nanosurface was obtained by two-step wet chemical treatment (5 M sodium hydroxide immersion and subsequent wet oxidization treatment in deionized water at 200°C). To produce surface Ca^2+^-modified nanostructured Ti samples (Nano/Ca surface), Ti disks were treated hydrothermally with a mixed solution of 0.1 M sodium hydroxide and 5 mM calcium oxide at 200°C for 2 h. After treatment, the samples were ultrasonically cleaned and air dried.

### 2.2. Surface Characterization

The surface nanotopography of the investigated samples was evaluated by atomic force microscopy ([AFM] XE-100; Park Systems, Suwon, Korea) using a 10 nm AFM tip in the noncontact mode (scan rate of 0.5 Hz). The surface roughness values of the samples were measured over a 2.5 *μ*m × 2.5 *μ*m scan area (*n* = 5) using the XEI image processing and analysis program (Park Systems). The crystalline structure of the surface oxide layer of the investigated samples was evaluated by thin-film X-ray diffractometry ([XRD] X'Pert-APD; Philips, Almelo, Netherlands). The chemical composition of the investigated surfaces was evaluated by X-ray photoelectron spectroscopy ([XPS] K-Alpha; Thermo Scientific, East Grinstead, UK). The surface wettability of Nanorough Ti (Nano and Nano/Ca surfaces) and bare Ti samples was evaluated using an automatic contact angle meter (Phoenix 3000; Surface Electro Optics, Seoul, Korea) by measuring the static contact angle (at 20 s) of one drop of deionized water (5 *μ*L) at room temperature (*n* = 8). In addition, the surface energy of investigated surfaces was automatically calculated following the Girifalco-Good-Fowkes-Young rule according to a previously described method [19].

### 2.3. Platelet Culture Experiments

#### 2.3.1. Preparation of Platelet-Rich Plasma

Whole blood drawn from a healthy volunteer was used to prepare platelet-rich plasma (PRP) by a double centrifugation technique according to the method described elsewhere [20, 21]. This study was approved by the Institutional Review Board of Kyungpook National University Hospital (Daegu, Republic of Korea; Approval no. KNUH 2015-09-019). Briefly, 3.8% citric acid was added to whole blood, followed by centrifugation at 160 g for 15 min to seperata the red blood cells. Then the upper plasma layer was transferred to a new tube and a second centrifugation was performed at 1500 g for 15 min. After centrifugation, supernatant containing platelet-poor plasma (PPP) layer was removed, and, finally, lower part of the plasma was used as the PRP. The number of platelets was measured with Neubauer hemocytometer counting chamber. The PPP was used to dilute the PRP. For the platelet culture experiments, the final platelet concentration in the PRP was adjusted to 1 × 10^5^ platelets/*μ*L PRP by adding PPP. Fresh PRP samples prepared within 2 h after blood sample collection were used for* in vitro* experiments. To evaluate the platelet response to Ti implant surfaces, 200 *μ*L of PRP activated by 2.5% calcium chloride solution was added to the surface of Ti samples.

#### 2.3.2. Morphological Evaluation of Platelet Adhesion and Fibrin Matrix Formation

Field emission-scanning electron microscopy (FE-SEM, S-4800; Hitachi, Tokyo, Japan) was used for the morphological evaluation of immediate platelet adhesion and fibrin matrix formation on the samples after 5 and 20 min of culture. At the indicated incubation time points, adherent platelets on Ti disks were sequentially fixed in 2% glutaraldehyde and 1% osmium tetroxide. After dehydration using an ascending series of alcohols and critical point drying and gold-palladium coating, fibrin matrix formation and adherent platelet morphology on the surfaces were observed.

#### 2.3.3. Enzyme-Linked Immunosorbent Assay for the Detection of Platelet-Derived Growth Factor Release

Platelet growth factor release was measured using a commercially available sandwich enzyme-linked immunosorbent assay (ELISA) kit (R&D systems, Minneapolis, MN, USA). Quantification of transforming growth factor-*β*1 (TFG-*β*1) and platelet-derived growth factor-AB (PDGF-AB) released from the PRP from the samples was conducted according to the manufacturer's instructions after 20 and 60 min of incubation. Briefly, the supernatant was collected and diluted 50-fold. Then, 100 *μ*L of the dilute solution was transferred to 96-well plates and the absorbance was measured at 450 nm with a microplate reader (*n* = 4 per group). For the quantification of TFG-*β*1, activation step was performed using 1 N HCl and then neutralized with 1.2 N NaOH and 0.5 M HEPES according to the manufacturer's instruction prior to prepare dilute solution. Concentrations of PDGF-AB and TGF-*β*1 released from the PRP on the investigated surfaces were calculated from the standard calibration curves generated by the standard solution of PDGF-AB and TGF-*β*1 prepared according to the manufacturer's instruction.

### 2.4. Macrophage Cell Culture Experiments

#### 2.4.1. Cell Culture

Mouse macrophage J774.A1 cells (American Type Culture Collection, Manassas, VA, USA) were maintained in Dulbecco's modified Eagle's medium containing 10% (v/v) fetal bovine serum, 100 U/mL penicillin, and 100 U/mL Fungizone under 100% humidity and 5% CO_2_, at 37°C.

#### 2.4.2. Immunofluorescent Staining for Evaluation of the Macrophage Phenotype Development

Immunocytochemistry was used to investigate early local expression levels of proinflammatory M1 marker (CCR7) and the proregenerative M2 marker (MR; CD206) in macrophage cells grown on the investigated surfaces. J774.A1 cells were seeded on Ti disk samples at an initial seeding density of 1 × 10^4^ cells/Ti disk. At 24 h of culture, cells adhered to Ti samples were fixed and permeabilized with 0.05% Triton X-100 (Thermo Fisher Scientific, Waltham, MA, USA) according to a previously described method [22]. Then, phenotype expression in macrophage cells double-stained using dilute solutions of primary antibodies (CCR7 [1:200 dilution, rabbit anti-mouse IgG; Abcam, Cambridge, MA, USA] and MR [1:100 dilution, rabbit anti-mouse IgG; Abcam]) and secondary antibodies (1:300 dilution, Alexa Fluor® 488 goat anti-rabbit IgG, Alexa Fluor® 555 goat anti-mouse IgG; Invitrogen, Carlsbad, CA, USA) were observed by confocal laser scanning microscopy ([CLSM] LSM700; Carl Zeiss, Oberkochen, Germany).

#### 2.4.3. ELISA for the Assessment of Inflammatory Cytokine Production

The concentrations of inflammation-related cytokines (tumor necrosis factor *α* [TNF*α*], interleukin 1*β* [IL1*β*], and prostaglandin E2 [PGE2]) secreted into culture media were measured with commercially available ELISA kits (R&D systems) at 24 h of culture (at an initial seeding density of 1 × 10^5^ cells/well) according to the manufacturer's instructions. Protein levels of proinflammatory cytokines in the supernatant sample were measured at 450 nm (*n* = 7 per group).

### 2.5. Statistical Analysis

Three independent cell culture experiments were performed. Statistical analysis was performed using one-way analysis of variance with Tukey's multiple comparison tests.* P *< 0.05 was considered statistically significant.

## 3. Results and Discussion

### 3.1. Surface Characteristics of the Samples

**Figure 1:**
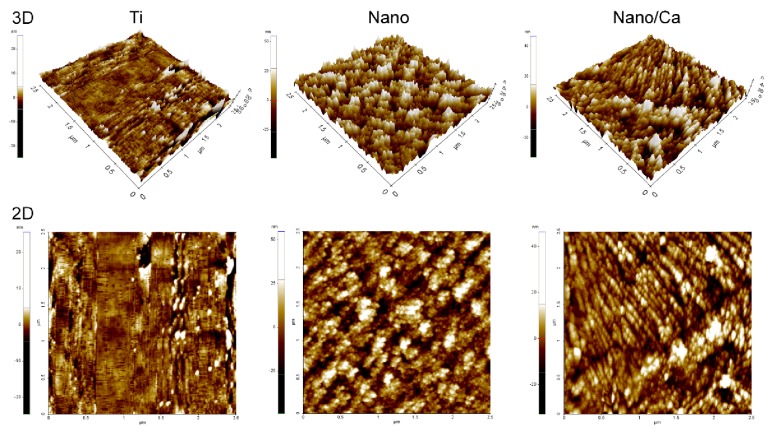
Representative 3D and 2D AFM images of the unmodified wet-abraded Ti, hydrothermally obtained pure nanostructured Nano and Ca^2+^-containing nanostructured Nano/Ca surfaces.

**Figure 2:**
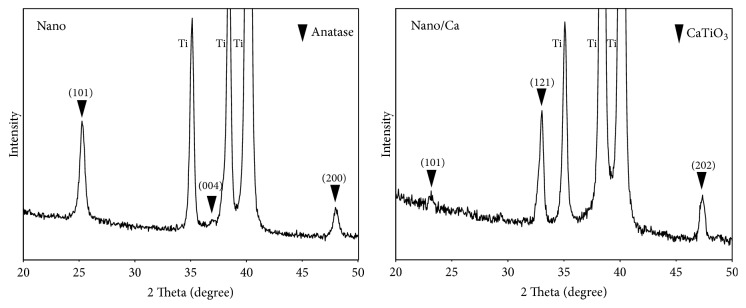
Thin-film X-ray diffraction patterns of the Nano and Nano/Ca samples.

Figure 1 shows representative two-dimensional (2D) and three-dimensional (3D) AFM images of the investigated surfaces. Relatively uniformly distributed nanostructures, tens of nanometers in width, covered surfaces of both the Nano and Nano/Ca samples. Surfaces of wet chemical-treated Nano and Nano/Ca samples were composed of a crystalline Ti oxide layer (Figure 2). Thin-film XRD analysis revealed formation of anatase titanium oxide (TiO_2_) structure (JCPDS #21-1272) in the Nanosample and a calcium titanate (CaTiO_3_) structure (JCPDS #22-0153) in the Nano/Ca sample (Figure 2).

**Figure 3:**
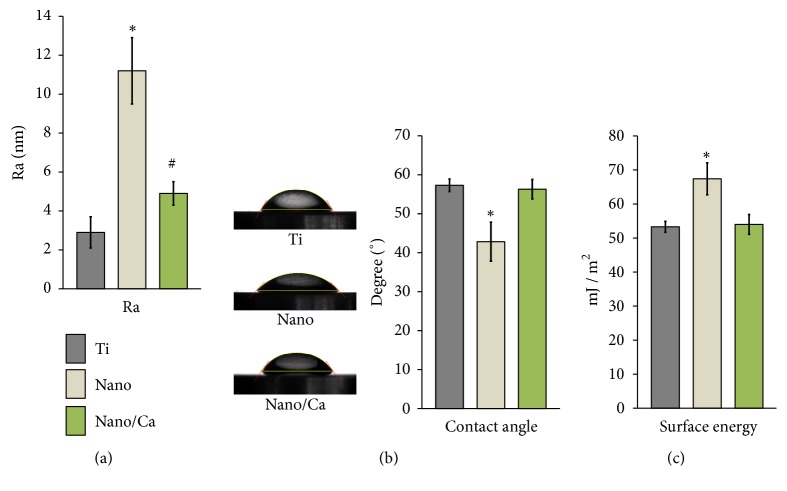
Surface characteristics of the investigated surfaces. (a)* Ra* values. (b) Water contact angles. (c) Surface energies. Data are presented as the mean ± SD. ^*∗*^*p* < 0.05 compared with the other surface; ^#^*p* < 0.05 between two surfaces.

The average surface roughness (*Ra*) values of the investigated surfaces were in a range of several nanometers (Ti and Nano/Ca samples) and up to 11 nm (Nanosample). The* Ra* value of the Nano and Nano/Ca surfaces was greater than that of the unmodified Ti surface (*p* < 0.05; Figure 3(a)). The Nanosurface had a significantly greater* Ra* value than that of both the Ti and Nano/Ca surfaces (*p* < 0.05).

The Ti sample with a surface anatase TiO_2_ structure and nanoscale topography (Nanosurface) exhibited a more hydrophilic surface property than the unmodified Ti and Ca^2+^-incorporated nanostructured (Nano/Ca) samples (Figure 3(b)). The Nanosurface exhibited a significantly lower water contact angle than the control Ti and Nano/Ca surfaces (*p* < 0.05; Figure 3(b)). The Nanosurface showed a significantly greater surface energy (67.4 ± 4.7 mJ/m^2^) than the Ti (53.3 ± 1.6 mJ/m^2^) and Nano/Ca (54 ± 2.9 mJ/m^2^) surfaces (*p* < 0.05; Figure 3(c)). There were no differences in water contact angle and surface energy between the unmodified Ti and Nano/Ca surfaces.

The surface chemical compositions of the investigated samples, as determined by XPS analysis, are shown in Table 1. The Nanosample had a relatively high percentage of surfaces Ti2p (22.2%) and O1s (55.4%) compared with the unmodified Ti and Nano/Ca samples. Ti samples obtained by wet chemical treatment using a Ca^2+^-containing solution had a relatively high percentage of surface Ca content. The atomic percentage of Ca2p for the Nano/Ca surface was 12.8% (Table 1).

### 3.2. Immediate Platelet Response

#### 3.2.1. Morphological Evaluation of Immediate Platelet Activation

**Figure 4:**
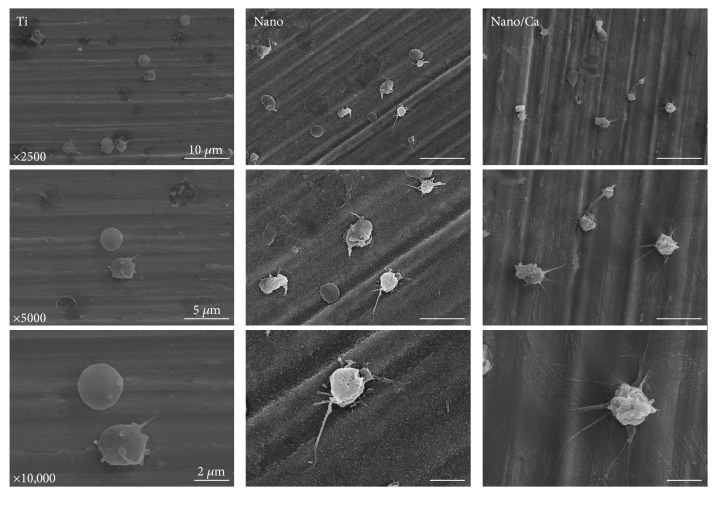
FE-SEM images showing the morphology of adherent blood platelets in the investigated samples after 5 min of incubation. Platelets in the Ti samples with surface nanostructure (Nano and Nano/Ca samples) and Ca^2+^ chemistry (Nano/Ca sample) had more dendritic spread than the unmodified Ti sample.

**Figure 5:**
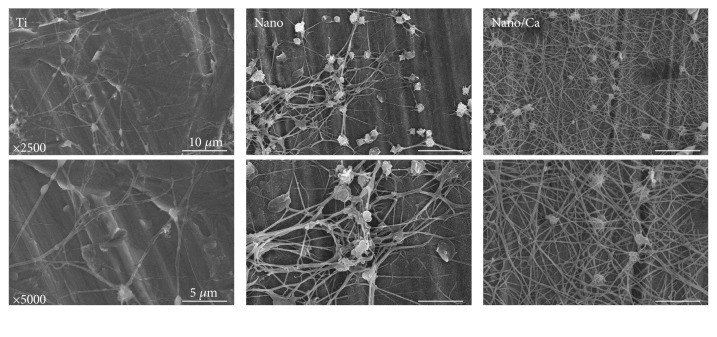
FE-SEM images showing the morphology of adherent platelets in the investigated samples after 20 min of incubation. Platelets at the Nano and Nano/Ca surfaces had denser fibrin matrix formation than the unmodified wet-abraded Ti sample.

The morphologies of adherent blood platelets on the investigated surfaces, as evaluated by FE-SEM, are shown in Figures 4 and 5. At 5 min of incubation, platelets adhering to all of the investigated surfaces had a round cell shape (Figure 4). However, platelets on the nanostructured surfaces (Nano and Nano/Ca) exhibited a more dendritic pattern than those on the unmodified Ti surface. Platelets on the Nano/Ca surface showed a tendency to have a more accentuated dendritic cell patten than those on the Nanosurface.

At 20 min, FE-SEM observation showed more evident clot formation patterns on all of the investigated surfaces than at 5 min of incubation (Figure 5). Formation of a mesh-like fibrin matrix was also observed in all of the investigated samples. The Nanosurface supported the formation of a denser network of fibrils with entrapped platelets compared with the unmodified Ti surface. Ca^2+^ incorporation further increased formation of denser fibrin matrix on the nanostructured Ti surface. These results suggest that surface nanotopographical modification is beneficial for inducing an immediate platelet response, which is favorable for the subsequent biological bone healing process, and surface Ca^2+^ modification increases platelet aggregation and fibrin network formation on the nanostructured Ti surface.

The dense fibrin network formed on implant surfaces serves as a temporary scaffold to promote homing of osteogenic MSCs to the implant surface [1, 2, 4, 17, 18]. Morphology changes in platelets adhering to the implant surface are indicative of stages of platelet activation [1, 2, 4, 17, 18, 23]. Platelet activation can be classified into three stages according to morphology changes: dendritic (early pseudopodia), spread dendritic (intermediate pseudopodia), and fully spread platelets [23]. The more advanced stage of platelet activation was present in the Nano/Ca sample, which was characterized by dense fibrin network formation and platelet spreading. These findings indicate that surface Ca^2+^ modification drives platelet activation by stimulating the interaction of microtubules and actin filaments through increasing internal Ca^2+^ concentration of platelets at the nanostructured surface [5, 23, 24]. We presume that Ca^2+^ release from the Nano/Ca sample was a possible reason for the enhanced platelet activation, which may have led to increased intracellular Ca^2+^ concentration and subsequent morphological alterations of platelets [11, 23].

#### 3.2.2. Platelet Growth Factor Production at the Investigated Surfaces

**Figure 6:**
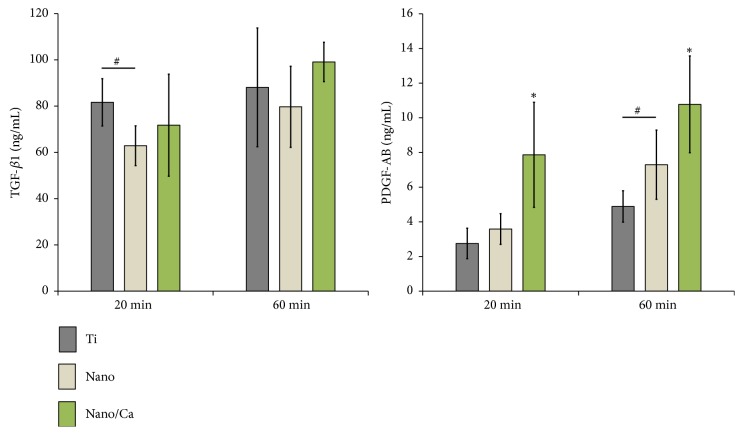
ELISA results for detection of TGF-*β*1 and PDGF-AB protein levels released from adherent platelets on the investigated surfaces after 20 and 60 min of incubation. Data are presented as the mean ± SD of three independent experiments. ^*∗*^*p* < 0.05 compared with the other surface; ^#^*p* < 0.05 between two surfaces.

After investigating platelet activation of the investigated samples by morphological evaluation using SEM observation, the concentrations of growth factors (TFG-*β*1 and PDGF-AB) released from activated platelets were evaluated by ELISA at 20 and 60 min of incubation. We were not able to find a significant correlation between TFG-*β*1 protein level and surface modification modality (Figure 6). At 20 min, platelets on the unmodified Ti surface showed greater TFG-*β*1 secretion compared with the Nanosurface, but no statistical differences were found in TFG-*β*1 protein level between the investigated surfaces at 60 min (Figure 6). In contrast, protein levels of PDGF-AB secretion tended to be greater in the nanostructured Ti samples (Nano and Nano/Ca) than the unmodified Ti sample (Figure 6). But no statistical difference was observed in PDGF-AB concentration between the control Ti and Nanosurfaces at 20 min of incubation (Figure 6). However, surface Ca^2+^-modified nanostructured Ti sample (Nano/Ca) exhibited significantly greater PDGF-AB release than the Ti and Nanosamples at 20 min of incubation (*p* < 0.05; Figure 6). At 60 min, protein levels of PDGF-AB secreted from adherent blood platelets were increased in all of the investigated surfaces when compared with 20 min of incubation. The pure nanostructured Ti surface had a significantly greater PDGF-AB concentration than the unmodified control Ti surface (*p* < 0.05; Figure 6). Surface Ca^2+^ modification increased PDGF-AB production in PRP compared with the pure Nano and unmodified flat Ti surfaces (*p* < 0.05).

TGF-*β*1 and PDGF-AB act as chemoattractant for MSCs and promote the proliferation of osteoblastic cells and angiogenesis [25–27]. Thus, more favorable growth factor release from activated blood platelets with modified Ti surface in the immediate early phase of implant healing would be beneficial for achieving a favorable osseointegration outcome. The release behavior of growth factors, such as TGF-*β*1 and PDGF-AB, is dependent on the degranulation of *α*-granules in activated platelets [3, 4, 13, 18]. But we were not able to find correlations between TFG-*β*1 production level and surface modification modalities in this study. Studies have shown variation in leukocyte content in PRP prepared by centrifugation [28–30]. For example, Everts* et al*. [28] reported variation in leukocyte expression in PRP prepared by centrifugation, with a concentration that was 1.3–3.1 times higher than that in whole blood. Leukocyte content strongly affects the TFG-*β*1 release profile in PRP [28]. Thus, we presume that dissimilar TFG-*β*1 release behavior in the PRP of the investigated Ti samples is attributable to variation of leukocyte contents in the PRP prepared by centrifugation and resultant TFG-*β*1 production by leukocytes [28–30]. However, we only suppose that different surface properties of modified Ti samples influenced leukocyte response and resultant TGF-*β*1 release in some way. On this, additional studies are needed for confirmation. In contrast, it was evident that the nanoscale surface topography and surface Ca^2+^ chemistry modification markedly increased platelet PDGF-AB release at the Ti implant surface.

Our results showed that surface chemistry modification with Ca^2+^ promoted platelet activation at the nanostructured Ti surface, with an effect that was superior to that of the pure anatase TiO_2_ structure with better surface hydrophilicity. These findings are somewhat in agreement with the results of other studies reporting that surface hydrophilicity, nanoscale topography, and crystalline TiO_2_ structure affect platelet activation on the Ti surface [2, 12–15]. The anatase TiO_2_ structure enhanced platelet activation, which accelerated dense fibrin matrix formation [13] and PDGF growth factor release [15]. Thus, increase of PDGF-AB release at the Nanosurface compared with the unmodified control Ti surface was attributable to the effects of nanoscale surface topography and the anatase TiO_2_ structure [13, 15]. Superior surface wettability of the Nanosample seemed to synergistically contribute to the immediate activation of platelets at the nanostructured Ti surface [12]. Thus, nanostructured Ti implant surface with enhanced surface hydrophilicity seems to be beneficial to enhance the initial stage healing process of implant osseointegration by promoting platelet activation. However, our results indicate that surface Ca chemistry (in this study, as in the form of CaTiO_3_ layer) surpasses the effect of surface hydrophilicity in promoting immediate blood platelet activation in the nanostructured Ti implant surface. In this study, surface Ca^2+^ modification also markedly promoted platelet activation at the nanostructured Ti surface, despite its lower surface hydrophilicity when compared with the Nanosurface. Together, these results indicate that surface Ca^2+^ modification may be beneficial for inducing early osteogenesis via promotion of the homing and osteogenic differentiation of MSCs by platelet activation at the nanostructured Ti implant surface.

### 3.3. Early Macrophage Response

#### 3.3.1. Macrophage M1 and M2 Phenotype Expression Assessed by Immunostaining

**Figure 7:**
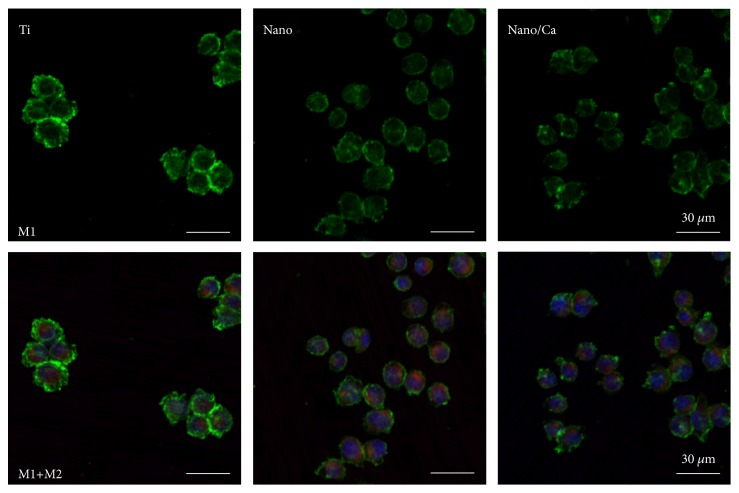
(upper panel) CLSM images showing expression of the proinflammatory M1 phenotype (CCR7, in green) in macrophages in the investigated samples (lower panel). Merged immunofluorescent images of macrophages expressing proinflammatory M1 and proregenerative M2 phenotypes (CD206, in red) in the investigated samples after 24 h incubation.

CLSM images of macrophage phenotype expression in J774.A1 cells induced by surface modification modalities are shown in Figure 7. At 24 h of culture, adherent J774.A1 macrophage cells on the unmodified Ti surface exhibited stronger local expression of the inflammation-related M1 phenotype (CCR7) compared with cells on the Nano and Nano/Ca surfaces. Macrophage cells on the Nano and Nano/Ca exhibited stronger M2 phenotype (MR; CD206) expression than on the control Ti surface (Figure 7). There were no notable differences in the immunostaining intensity of the M1 and M2 phenotypes (i.e., CCR7 and CD206) between the Nano and Nano/Ca samples.

#### 3.3.2. Inflammation-Related Cytokine Production by Adherent Macrophage Cells

**Figure 8:**
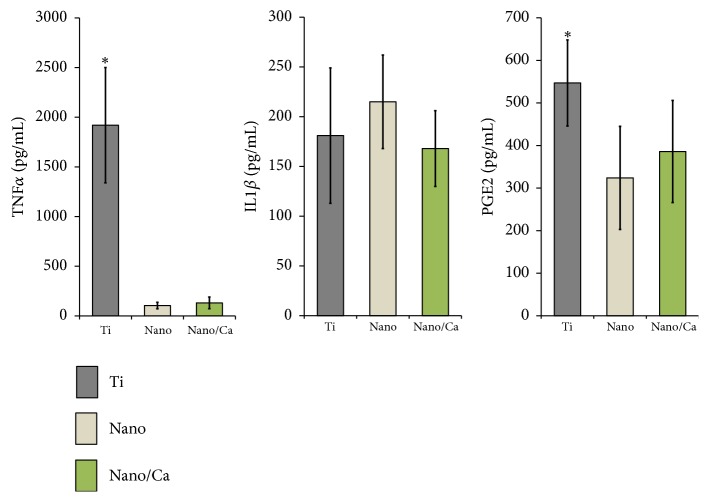
ELISA results of TNF*α*, IL1*β*, and PGE2 levels secreted into the culture media by adherent J774.A1 macrophages on the investigated surface after a 24 h incubation. Data are presented as the mean ± SD of three independent experiments. ^*∗*^*p* < 0.05 compared with the other surface.

Early protein production levels of proinflammatory cytokines (TNF*α*, IL1*β*, and PGE2) in macrophage cells grown on the investigated surfaces are shown in Figure 8. At 24 h of incubation, macrophage cells grown on the unmodified Ti surface secreted markedly higher levels of TNF*α* into culture media compared with the Nano and Nano/Ca surfaces (15- to 18-fold greater,* p* < 0.05). However, no difference was found in the TNF*α* protein levels between the Nano and Nano/Ca surfaces. There was also no difference in IL1*β* production levels between any of the investigated samples (Figure 8). Early PGE2 production by adherent macrophage cells was increased at the unmodified Ti surface compared with the Nano and Nano/Ca surfaces.

These findings indicate that both the surface nanotopographical and Ca^2+^ modifications suppress inflammatory M1 macrophage phenotype development at the Ti implant surface in the early healing stage. Positive modulation of macrophage phenotype expression by surface nanostructure and Ca^2+^ modification at this stage may induce subsequently favorable osteogenesis outcome regulated by osteogenic MSCs at the implant surface [7, 25, 26, 31, 32].

## 4. Conclusions

In this study, we investigated the effects of nanoscale surface topographical and Ca^2+^ modifications on the immediate and early responses of platelets and macrophages at the Ti implant surface. Understanding the biological mechanism underlying the bone healing capacity of modified Ti implants in the initial phase of healing (i.e., proceeding the osteogenic cell-governed healing phase) is essential for the future surface design of Ti bone implants with enhanced osteogenic capacity. Our results demonstrate that nanotopographical surface modification of Ti accelerates platelet activation, which promotes fibrin matrix formation and PDGF-AB release. Surface Ca^2+^ modification enhanced immediate platelet activation at the nanostructured Ti surface. Surface nanotopographical and Ca^2+^ modifications strongly suppressed inflammation-related macrophage phenotype at an early incubation time, which in turn significantly decreased production of the inflammatory cytokines, TNF*α* and PGE2, in macrophage cells at the Ti surface. These findings suggest that surface nanotopography and Ca^2+^ chemistry positively modulate the immediate and early phase healing of bone healing around the Ti implants, which is regulated by platelets and macrophages. This may be an important mechanism underlying the enhanced osteogenic capacity of modified Ti implants.

## Figures and Tables

**Table 1:** Chemical composition of investigated surfaces by X-ray photoelectron spectroscopy (atomic %).

Group	Ti2p	O1s	C1s	Ca2p	Na1s	N1s
Ti	18.4	53.7	25.1		1.7	1.1
Nano	22.2	55.4	21.7		< 0.1	0.7
Nano/Ca	12.2	51.3	22.1	12.8	0.8	0.8

## Data Availability

The data used to support the findings of this study are available from the corresponding author upon request.

## References

[1] Park J. Y., Gemmell C. H., Davies J. E. (2001). Platelet interactions with titanium: modulation of platelet activity by surface topography. *Biomaterials*.

[2] Huang H.-H., Chen J.-Y., Lin M.-C., Wang Y.-T., Lee T.-L., Chen L.-K. (2012). Blood responses to titanium surface with TiO 2 nano-mesh structure. *Clinical Oral Implants Research*.

[3] Anitua E., Zalduendo M. M., Alkhraisat M. H., Orive G. (2013). Release kinetics of platelet-derived and plasma-derived growth factors from autologous plasma rich in growth factors. *Annals of Anatomy*.

[4] Alfarsi M. A., Hamlet S. M., Ivanovski S. (2015). The Effect of Platelet Proteins Released in Response to Titanium Implant Surfaces on Macrophage Pro-Inflammatory Cytokine Gene Expression. *Clinical Implant Dentistry and Related Research*.

[5] Milleret V., Tugulu S., Schlottig F., Hall H. (2011). Alkali treatment of microrough titanium surfaces affects macrophage/monocyte adhesion, platelet activation and architecture of blood clot formation. *European Cells and Materials*.

[6] Champagne C. M., Takebe J., Offenbacher S., Cooper L. F. (2002). Macrophage cell lines produce osteoinductive signals that include bone morphogenetic protein-2. *Bone*.

[7] Brown B. N., Ratner B. D., Goodman S. B., Amar S., Badylak S. F. (2012). Macrophage polarization: an opportunity for improved outcomes in biomaterials and regenerative medicine. *Biomaterials*.

[8] Ma Q.-L., Zhao L.-Z., Liu R.-R. (2014). Improved implant osseointegration of a nanostructured titanium surface via mediation of macrophage polarization. *Biomaterials*.

[9] Galli S., Naito Y., Karlsson J. (2014). Local release of magnesium from mesoporous TiO2 coatings stimulates the peri-implant expression of osteogenic markers and improves osteoconductivity in vivo. *Acta Biomaterialia*.

[10] Wang G., Li J., Zhang W. (2014). Magnesium ion implantation on a micro/nanostructured titanium surface promotes its bioactivity and osteogenic differentiation function. *International Journal of Nanomedicine*.

[11] Kim H.-S., Kim Y.-J., Jang J.-H., Park J.-W. (2016). Surface Engineering of Nanostructured Titanium Implants with Bioactive Ions. *Journal of Dental Research*.

[12] Alfarsi M. A., Hamlet S. M., Ivanovski S. (2014). Titanium surface hydrophilicity enhances platelet activation. *Dental Materials*.

[13] Huang Q., Yang Y., Hu R., Lin C., Sun L., Vogler E. A. (2015). Reduced platelet adhesion and improved corrosion resistance of superhydrophobic TiO2-nanotube-coated 316L stainless steel. *Colloids and Surfaces B: Biointerfaces*.

[14] Huang Q., Yang Y., Zheng D. (2017). Effect of construction of TiO2 nanotubes on platelet behaviors: Structure-property relationships. *Acta Biomaterialia*.

[15] Zhang L., Liao X., Fok A., Ning C., Ng P., Wang Y. (2018). Effect of crystalline phase changes in titania (TiO 2 ) nanotube coatings on platelet adhesion and activation. *Materials Science and Engineering C: Materials for Biological Applications*.

[16] Sawada R., Kono K., Isama K., Haishima Y., Matsuoka A. (2013). Calcium-incorporated titanium surfaces influence the osteogenic differentiation of human mesenchymal stem cells. *Journal of Biomedical Materials Research Part A*.

[17] Gupta S., Reviakine I. (2012). Platelet activation profiles on TiO2: effect of Ca2+ binding to the surface.. *Biointerphases*.

[18] Tejero R., Rossbach P., Keller B., Anitua E., Reviakine I. (2013). Time-of-flight secondary ion mass spectrometry with principal component analysis of titania-blood plasma interfaces. *Langmuir*.

[19] Park J.-W., Kim Y.-J., Park C. H. (2009). Enhanced osteoblast response to an equal channel angular pressing-processed pure titanium substrate with microrough surface topography. *Acta Biomaterialia*.

[20] Tanaka Y., Kurashima K., Saito H. (2009). In vitro short-term platelet adhesion on various metals. *The International Journal of Artificial Organs*.

[21] Dhurat R., Sukesh M. (2014). Principles and methods of preparation of platelet-rich plasma: a review and author's perspective. *Journal of Cutaneous and Aesthetic Surgery*.

[22] Lee C.-H., Kim Y.-J., Jang J.-H., Park J.-W. (2016). Modulating macrophage polarization with divalent cations in nanostructured titanium implant surfaces. *Nanotechnology*.

[23] Matarrese P., Straface E., Palumbo G. (2009). Mitochondria regulate platelet metamorphosis induced by opsonized zymosan A - Activation and long-term commitment to cell death. *FEBS Journal*.

[24] Nesbitt W. S., Giuliano S., Kulkarni S., Dopheide S. M., Harper I. S., Jackson S. P. (2003). Intercellular calcium communication regulates platelet aggregation and thrombus growth. *The Journal of Cell Biology*.

[25] Ogino Y., Ayukawa Y., Kukita T., Koyano K. (2006). The contribution of platelet-derived growth factor, transforming growth factor-*β*1, and insulin-like growth factor-I in platelet-rich plasma to the proliferation of osteoblast-like cells. *Oral Surgery, Oral Medicine, Oral Pathology, Oral Radiology, and Endodontology*.

[26] Ponte A. L., Marais E., Gallay N. (2007). The in vitro migration capacity of human bone marrow mesenchymal stem cells: comparison of chemokine and growth factor chemotactic activities. *Stem Cells*.

[27] Ng F., Boucher S., Koh S. (2008). PDGF, TGF-beta, and FGF signaling is important for differentiation and growth of mesenchymal stem cells (MSCs): transcriptional profiling can identify markers and signaling pathways important in differentiation of MSCs into adipogenic, chondrogenic, and osteogenic lineages. *Blood*.

[28] Everts P. A. M., Hoffmann J., Weibrich G. (2006). Differences in platelet growth factor release and leucocyte kinetics during autologous platelet gel formation. *Transfusion Medicine*.

[29] Dohan Ehrenfest D. M., Bielecki T., Jimbo R. (2012). Do the fibrin architecture and leukocyte content influence the growth factor release of platelet concentrates? An evidence-based answer comparing a pure Platelet-Rich Plasma (P-PRP) gel and a leukocyte- and Platelet-Rich Fibrin (L-PRF). *Current Pharmaceutical Biotechnology*.

[30] Roh Y. H., Kim W., Park K. U., Oh J. H. (2016). Cytokine-release kinetics of platelet-rich plasma according to various activation protocols. *Bone & Joint Research*.

[31] Terheyden H., Lang N. P., Bierbaum S., Stadlinger B. (2012). Osseointegration—communication of cells. *Clinical Oral Implants Research*.

[32] Vanden Berg-Foels W. S. (2014). In situ tissue regeneration: Chemoattractants for endogenous stem cell recruitment. *Tissue Engineering - Part B: Reviews*.

